# Risk and resilience in the red lights: a mini-review on sex worker lived experiences and mental health outcomes

**DOI:** 10.3389/fpubh.2026.1773690

**Published:** 2026-02-25

**Authors:** Logan Blouin, Ellis Sather, Anna Bowlin

**Affiliations:** Department of Counseling, Loyola University New Orleans, New Orleans, LA, United States

**Keywords:** sex work, mental health, stigma, occupational autonomy, community resilience, intersectional vulnerability, masculine sexual entitlement

## Abstract

Sex workers experience disproportionately high rates of depression, anxiety, PTSD, suicidality, dissociation, and substance use across global contexts. Sex work refers to the consensual exchange of sexual services, performances, or content for compensation; debates persist regarding the impact of coercion and constraint within sex work, particularly in relation to structural and economic pressures. This mini-review synthesizes research on the structural, social, and occupational determinants of sex workers’ mental health, emphasizing how legal frameworks, work venues, stigma, and interpersonal dynamics shape psychological outcomes. Sex work occurs across indoor, outdoor, and digital settings, with workers often moving fluidly between contexts; mental health risk consistently increases in criminalized environments and in settings characterized by reduced safety and autonomy. Drawing on Goffman’s stigma framework, the review examines external and internalized stigma as central mechanisms linking criminalization, discrimination, and healthcare avoidance to psychological distress, with compounded effects for LGBTQIA+ and BIPOC sex workers. Particular attention is given to masculine sexual entitlement as a proximal interpersonal stressor strongly predictive of PTSD symptoms. Across studies, violence exposure, poverty, early entry into sex work, and low workplace control are associated with elevated mental health burden, whereas peer and community networks function as robust protective factors that enhance safety, autonomy, and access to care. Implications include sex work-affirming, trauma-informed clinical care and community-partnered research.

## 1 Introduction

### 1.1 Structural, social, and occupational contexts of sex work

Sex work is often categorized as indoor (e.g., clubs, brothels, hotels, private residences) or outdoor/street-based work in public settings (e.g., vehicles, parks, truck stops) (29). Digital platforms (e.g., OnlyFans) have expanded opportunities for autonomy and income while introducing distinct forms of stigma, surveillance, and privacy risk (29). Many workers move across settings based on safety, clientele, and financial need, making these categories fluid rather than fixed (29).

Globally, sex work is regulated under four primary legal models: (a) full criminalization, (b) partial criminalization/end-demand (Nordic), (c) legalization through state regulation (e.g., licensing/registration), and (d) decriminalization, which removes sex-work-specific criminal penalties and treats sex work as labor under general workplace and consent laws (1–3). Across studies, mental health risk often increases with reduced safety and autonomy over working conditions (4). Street-based workers experience elevated violence risk, often perpetrated by clients, law enforcement, or intimate partners (4). Criminalized environments constrain safety strategies, such as client screening, working indoors or with peers, and negotiating terms openly, pushing workers into more dangerous conditions for fear of being arrested, and restricting sex workers from reporting crimes committed against them such as assault or robbery (5). Police harassment and abuse are also widely reported, and exposure to police interaction predicts mental health distress (4). However, meta-analytic findings suggest legal status alone may not consistently predict mental health burden (6). These differences may reflect variation in enforcement intensity, access to harm reduction resources, and the extent to which workers can implement safety strategies.

#### 1.1.1 Digital sex work contexts

Digital sex work can involve intensified surveillance and legal vulnerability. Jones (7) argues that online workers face heightened monitoring under policies shaped by prejudice, highlighting FOSTA/SESTA as contributing to increased surveillance and disproportionate impacts on marginalized sex workers. Online harms such as doxxing (exposure of legal identity, address, workplace) further blur boundaries between labor and private life, producing ongoing fear, hypervigilance, and psychological distress (7).

#### 1.1.2 Whorearchy: diversity and hierarchies of labor

Sex work operates within an informal hierarchy (“whorearchy,” a term used colloquially within sex workers’ communities and some qualitative research) shaped by working conditions, “respectability,” visibility, proximity to clients, and criminalization (8–10). Online and indoor arrangements (e.g., sugar dating) are often framed as more socially acceptable than street-based or survival work (sex work in exchange for food, housing, or safety), which remains heavily stigmatized (8, 10). This stratification mirrors dominant cultural values (e.g., white, middle-class, feminine norms), compounding stigma for workers who are racialized, poor, or unhoused. This status differential may also reflect material working-condition differences across settings, including variations in autonomy over working conditions, income, and exposure to violence (6). Overall, sex work is best understood as a heterogeneous labor landscape shaped by intersecting legality, inequality, and stigma.

## 2 Stigma in sex work

Using Goffman’s (11) stigma theory, this mini-review conceptualizes stigma as a social process that marks individuals as “tainted” or “deviant” through culturally defined standards of morality and respectability. In sex work, stigma reinforces hierarchies of gender, sexuality, race, and class through structural and interpersonal discrimination, psychological impacts, and barriers to care (12).

Across studies, stigma functions as a core driver of mental health outcomes by shaping self-concept, restricting access to services, and converting external discrimination into internal distress (3, 4, 13–15). In this sense, stigma both produces and sustains depression, anxiety, trauma, and suicidality among sex workers (1, 6, 16).

Stigma is compounded by intersecting marginalized identities. LGBTQIA+ sex workers—particularly transgender and gender-expansive individuals—face additional stigma (transphobia/homophobia) that intensifies violence and discrimination (7). Jones (7) also found that BIPOC sex workers’ racialized hypersexualization is linked to heightened police surveillance, public harassment, and structural exclusion. Consistent with setting- based hierarchies, stigma is contextually stratified and reflects broader social biases around gender, class, and race.

### 2.1 External stigma: discrimination, criminalization, and service avoidance

External stigma includes overt discrimination, exclusion, and judgment in institutional and social settings (4, 17). In Western media contexts, narratives frequently portray sex workers as deviants, criminals or inherently victimized, reinforcing stereotypes that legitimize punitive responses (5). In a global review, most participants reported discrimination when seeking healthcare or law enforcement support, contributing to avoidance of essential services and increasing vulnerability to adverse outcomes (4).

### 2.2 Internalized stigma and self-concept

Internalized stigma describes the incorporation of societal devaluation into self-blame, shame, and concealment, which is associated with depression, anxiety, and trauma-related symptoms (4). Bennett-Brown et al. (18) found that internalized stigma shifts between harm and resistance depending on social support and autonomy over working conditions, suggesting that stigma’s psychological impact is shaped by relational and structural context.

## 3 Mental health outcomes among sex workers

Across geographic, legal, and work contexts, sex workers report markedly higher rates of mental health problems than non-sex-working populations (4, 6, 16). Because sex work is highly feminized (nearly 90%), sex worker prevalence estimates are often compared to global female baselines when available (4). Although research frequently centers cisgender women, evidence suggests that workers with intersecting minority identities (e.g., BIPOC, LGBTQIA+, disabled, undocumented, transgender, non-binary, and male sex workers) experience equal or greater mental health burdens (7).

### 3.1 Prevalence of depression, PTSD, anxiety, and suicidality

Meta-analyses report that 19–88% of sex workers meet clinical criteria for depression, compared with 6.9% of women globally (30). PTSD prevalence among sex workers ranges from 29 to 75%, compared with roughly 5% among women in the general population (6, 16, 28). Anxiety disorders range from 13 to 63% among sex workers, exceeding the 4.4% baseline for women (4, 19, 31). Suicidal ideation ranges from 12 to 38%, compared with 9% in the general population (14, 16, 32). Researchers attribute these disparities primarily to stigma, criminalization, violence, and social exclusion rather than sex work *per se* (1), while also noting likely underreporting due to stigma and recruitment barriers (3, 4).

### 3.2 Dissociation and substance use

Dissociative symptoms are common, including memory problems (68%), flashbacks (65%), derealization (59%), and depersonalization (50%), with a meaningful subset meeting dissociative disorder criteria (20). Substance use disorders are also prevalent: across meta-analyses, 10–73% of sex workers report substance use disorder symptoms at some point in their lives (4, 6, 16). Substance use can function both as a response to distress and a factor that intensifies it, contributing to reinforcing cycles of harm (4, 6, 16). Alcohol and stimulant use are frequently reported and often comorbid with mood and anxiety problems (6, 21).

### 3.3 Other mental health challenges and somatic burden

Beyond depression, PTSD, anxiety, and substance use, studies describe burnout, emotional exhaustion, and compassion fatigue linked to prolonged emotional labor, stigma exposure, and unsafe environments (1, 22, 23). Chronic stress is often tied to criminalization, poverty, and structural insecurity (4, 6). Some studies also report personality-related distress (e.g., impulsivity, emotional dysregulation) and common somatic symptoms (chronic pain, fatigue, sleep disturbance), reflecting the physiological toll of sustained psychosocial stress (19, 24, 25). Comorbidity is typical: clusters of depression, PTSD, anxiety, suicidality, and substance use frequently co-occur, consistent with cumulative and interacting stressors (4, 16).

## 4 Structural and interpersonal determinants of mental health outcomes

Poor mental health is consistently linked to structural forces (criminalization, poverty, violence, healthcare barriers) and work-context factors (venue, working conditions, autonomy) (4, 6, 15, 16, 21, 24, 27). Across studies, stigma underlies and amplifies these risks (3, 4).

### 4.1 Masculine sexual entitlement

Masculine sexual entitlement functions as a distinct mechanism within stigma. Raines (26) describes six themes: (a) prioritizing sexual needs of self, (b) objectification of others, (c) peer norms, (d) essentialist gender attitudes, (e) sexual deception and pressure, and (f) minimizing or dismissing problematic behavior (p. 46). This entitlement frames women’s bodies—especially those in sexual labor—as accessible and violable, normalizing boundary violations and undermining autonomy. Empirically, perceived client entitlement predicts harm: Alschech et al. (13) found it was the strongest predictor of PTSD symptoms, exceeding documented sexual or physical violence. Sex workers report that chronic expectation to tolerate fear, discomfort, unwanted touch, or coercion fosters persistent hypervigilance and emotional exhaustion (6, 13, 16).

### 4.2 Criminalization, poverty, autonomy, and venue

Criminalization is a strong structural predictor of distress. Sex workers in criminalized or partially criminalized contexts report substantially higher PTSD and depression, attributed to fear of arrest, police abuse, and lack of legal recourse (16, 21, 27). Poverty and economic insecurity predict elevated PTSD and anxiety and erode safety and access to care (4, 14). Workplace autonomy is similarly protective: limited decision-making power predicts higher depression and PTSD-related symptoms, while more choice over working conditions is correlated to better depression and PTSD symptom outcomes (17, 22). Violence is pervasive and strongly linked to trauma outcomes; in one meta-analytic synthesis, 61% reported sexual or physical violence, with PTSD far higher among those exposed (16). Venue moderates risk: PTSD prevalence is higher among street-based workers than among indoor/brothel workers, and outdoor work is associated with higher depression and PTSD due to reduced safety and environmental control (6, 16). Early entry (before age 18) is associated with higher PTSD and depression, often linked to coercion, unstable housing, and prolonged exposure to adversity (4, 16). Collectively, these determinants indicate that distress is a predictable consequence of structural inequities and occupational precarity rather than individual pathology (1, 3, 4, 6, 13).

### 4.3 Protective processes and resilience: community

Social isolation is consistently associated with distress; higher loneliness and weaker peer support predict greater depressive, anxiety, and somatic symptom severity (3, 19). In contrast, peer and community supports buffer the effects of stigma, criminalization, poverty, and violence (4, 6, 15, 16). Reliable social networks reduce isolation, increase perceived safety, and correlate with lower PTSD, depression, and anxiety across contexts (4, 6). Family cohesion and perceived safety at home similarly predict reduced depressive and anxious symptoms (14).

Peer networks often function as informal mental-health systems by offering emotional support, safety coordination, and navigation of services, promoting help-seeking in contexts where stigma and criminalization constrain disclosure (4, 6, 15). Community participation strengthens coping and self-regulation and is associated with lower depression, anxiety, somatic distress, and substance-use symptoms (14, 19). Within workplaces, strong peer relationships support collective safety strategies (e.g., screening, shared protocols), which are linked to lower PTSD relative to isolated working conditions (6, 16).

## 5 Discussion

### 5.1 Limitations and directions for future research

Research on sex workers’ mental health remains constrained by access barriers related to stigma, criminalization, and mobility, resulting in small, convenience-based samples that may not represent population diversity (16, 24, 25). Intersectional factors (race, gender identity, disability, neurodivergence, sexual orientation, immigration status, class) are often undermeasured or not analyzed as moderators/mediators, limiting explanatory power (13, 24). Reliance on self-report instruments is common and may be vulnerable to underreporting and cultural variation in symptom expression without diagnostic or qualitative triangulation (4, 13, 16). Finally, many studies reflect top-down epistemologies that risk deficit framing and insufficient consultation with the communities studied; peer-led and participatory designs remain uncommon despite their benefits for trust and validity (15). Future work should prioritize inclusive, community-centered, methodologically diverse research and evaluate trauma-informed, harm-reduction, and peer-led interventions (13, 15, 16, 24). Peer-led or co-researcher models are recommended to strengthen relevance, recruitment, and interpretation (15).

### 5.2 Counselor implications

Provider stigma is a major barrier: 79% of sex workers reported avoiding mental health services after stigmatizing encounters (15), and healthcare avoidance is associated with worse anxiety and chronic depressive symptoms (2, 14). Clinicians must provide non-stigmatizing care and pursue training informed by sex workers’ expertise, including education, visible professional support, and consultation networks focused on sex-worker-affirming practice. Given common presenting concerns, competencies in trauma, mood and anxiety disorders, dissociation, substance use, and intersectionality are especially relevant (16). Clinicians should also attend to masculine sexual entitlement as a predictor of PTSD and consider broader therapeutic approaches that address entitlement norms (13). Where possible, practitioners should rely on research conducted with meaningful sex worker involvement to reduce bias and improve clinical fit (16).

## 6 Conclusion

Overall, evidence consistently documents elevated depression, PTSD, anxiety, suicidality, dissociation, and substance use among sex workers, largely shaped by stigma, criminalization, violence, and structural exclusion rather than sex work itself. The field remains limited by sampling constraints, heavy self-report reliance, and insufficient intersectional analysis. Progress requires research and practice that are trauma-informed, community-centered, and grounded in sex workers’ agency and lived expertise to advance mental health equity for those engaged in sexual labor (13, 15, 16, 24).

## Author’s note

The authors approach this work with a reflexive awareness of how lived experience, professional training, and social context shape research. One or more authors have lived experience as sex workers, informing sensitivity to lived experience, stigma, structural harm, and gaps in the existing literature. The manuscript was developed through collaborative academic review and grounded entirely in peer-reviewed literature to ensure analytic rigor and minimize bias.
